# Coagulation parameters abnormalities and their relation to clinical outcomes in hospitalized and severe COVID-19 patients: prospective study

**DOI:** 10.1038/s41598-022-16915-8

**Published:** 2022-08-01

**Authors:** Hend M. Esmaeel, Heba A. Ahmed, Mahmoud I. Elbadry, Asmaa R. Khalaf, Nesreen A. Mohammed, Hamza A. Mahmoud, Elhaisam M. Taha

**Affiliations:** 1 Department of Chest Disease and Tuberculosis, Faculty of Medicine, Sohag University, Sohag, 82524 Egypt; 2 Department of Clinical and Chemical Pathology, Faculty of Medicine, Sohag University, Sohag, Egypt; 3 Division of Haematology, Department of Internal Medicine, Faculty of Medicine, Sohag University, Sohag, Egypt; 4 Department of Public Health and Community Medicine, Faculty of Medicine, Sohag University, Sohag, Egypt; 5 Department of Anesthesia and Critical Care, Faculty of Medicine, Sohag University, Sohag, Egypt

**Keywords:** Prognostic markers, Medical research

## Abstract

There has been growing attention toward the predictive value of the coagulation parameters abnormalities in COVID-19. The aim of the study was to investigate the role of coagulation parameters namely Prothrombin concentration (PC), activated Partial thromboplastin Time (aPTT), D-Dimer (DD), Anti Thrombin III (ATIII) and fibrinogen (Fg) together with hematological, and biochemical parameters in predicting the severity of COVID-19 patients and estimating their relation to clinical outcomes in hospitalized and severe COVID-19 Patients. In a prospective study, a total of 267 newly diagnosed COVID-19 patients were enrolled. They were divided into two groups; hospitalized group which included 144 patients and non-hospitalized group that included 123 patients. According to severity, the patients were divided into severe group which included 71 patients and non-severe group that included 196 patients who were admitted to ward or not hospitalized. Clinical evaluation, measurement of coagulation parameters, biochemical indices, outcome and survival data were recorded. Hospitalized and severe patients were older and commonly presented with dyspnea (P ≤ 0.001). Differences in coagulation parameters were highly significant in hospitalized and severe groups in almost all parameters, same for inflammatory markers. D-dimer, AT-III and LDH showed excellent independently prediction of severity risk. With a cut-off of > 2.0 ng/L, the sensitivity and specificity of D dimer in predicting severity were 76% and 93%, respectively. Patients with coagulation abnormalities showed worse survival than those without (p = 0.002). Early assessment and dynamic monitoring of coagulation parameters may be a benchmark in the prediction of COVID-19 severity and death.

## Introduction

Severe Acute respiratory coronavirus 2 (SARS-CoV-2) is the virus that caused corona virus disease (COVID-19) that emerged as a public health major threat^1^. The disease prognosis is not constant. Mostly good, yet some cases exhibit progression to severe or critical care illness with coagulation dysfunction, acute respiratory distress syndrome and organ dysfunction^2^.

There has been growing attention toward the predictive value of the coagulation parameters abnormalities in COVID-19.

The derangements in the coagulation variables in COVID-19 patients and their prognostic value became research potentials^3^. Monitoring coagulation variables became an integral component of disease management as it may require additional interventions, and it can be associated with unwanted outcomes^4^.

The aim of the study was to investigate role of coagulation parameters namely prothrombin concentration (PC), activated partial thromboplastin time (aPTT), D-Dimer (DD), anti-thrombin III (ATIII) and fibrinogen (Fg) together with hematological, and biochemical parameters in predicting the severity of COVID-19 patients and estimating their relation to clinical outcomes in hospitalized and severe COVID-19 Patients.

## Methods

In a prospective hospital based study, a total of 267 newly diagnosed COVID-19 patients were enrolled in the study during the period from January 2021 to June 2021.

### Inclusion criteria

Adult (> 18 years) COVID-19 patients. The diagnosis of COVID-19 was done according to the World Health Organization interim guidance^5^.

Patients were excluded if they had pre-existing diseases resulting in abnormal coagulation parameters or received any drugs affecting hemostasis before inclusion in the study. Bed ridden patients, pregnant women, patients with liver disease, kidney disease or cancer were also excluded from the study.

The severity of the disease was assessed according to the NIH COVID-19 Treatment Guidelines^6^. This guideline group SARS-CoV-2 infection into five categories based on severity of illness; Asymptomatic or pre-symptomatic infection, mild illness, moderate illness, severe illness and critical illness.

In our study, patients with moderate, severe and critical care illness were hospitalized. The hospitalized group included 144 patients and the non-hospitalized group included 123 patients. In hospital those with severe and critical illness were admitted to ICU and considered as severe study group (71 patients), This included patients people who have oxygen saturation < 94% on room air at sea level, a ratio of arterial partial pressure of oxygen to fraction of inspired oxygen (PaO2/FiO2) < 300 mm Hg, a respiratory rate > 30 breaths/min, or lung infiltrates > 50% or people who have respiratory failure, septic shock, and/or multiple organ dysfunction. Those with moderate illness were admitted to ward (73 patients) and together with the non -hospitalized patients (123 patients) considered as non –severe study group (196 patients).

All patients were subjected to history taking with assessment of the presenting symptoms, physical examination and laboratory investigations as follow:

- Complete blood count was performed on EDTA samples XN-1000 (Sysmex, Japan). The neutrophil–lymphocyte ratio (NLR) which is the relative number of neutrophils divided by the relative number of lymphocytes was calculated. We used the NLR as an indicator of systemic inflammation which was used frequently as a guide for the prognosis of various diseases, such as cancer, community pneumonia, and sepsis^7^.
- Coagulation parameters were measured as follow: blood is mixed with 109 mmol/L sodium citrate for anticoagulation (sodium citrate: blood = 1:9), The blood samples were centrifuged at 3000 rpm for 10 min within 1 h of collection to obtain platelet-poor plasma.

The activated partial thromboplastin time (aPTT), prothrombin concentration (PC) and fibrinogen (Fg) were analyzed by clotting methode. Antithrombin III (AT III) was analyzed by the chromogenic substrate assay. The D-dimer content was measured by immunoturbidimetric assay using automatic blood coagulation analyzer and reagents purchased Sysmex CS-1600 System (Sysmex corporation, Kobe, Japan).

- The biochemical indices: lactate dehydrogenase (LDH) and ferritin were measured using C501 (La Roche Ltd, Risch-Rotkreuz, Switzerland).
- Diagnosis of covid 19 infection was made with real time RT-PCR from nasopharyngeal swab.
- Erythrocyte sedimentation rate (ESR) was estimated by Westergren method.
- C‑reactive protein (CRP) was estimated by Latex agglutination test provided by Reactivos GPL Barcelona Spain.

All blood samples were collected within 2 h of admission and before starting any medication to avoid drug interference with lab testing. Blood testing on admission was only recorded. The patient’s venous blood was drawn by trained, qualified phlebotomists. If several measurements were available on the day of enrollment in the study for same patients, the first one at the time point of diagnostic criteria fulfillment was always used to maximize consistency among the patient population.

The patients were categorized according to the presence or absence of abnormalities in coagulation parameters into patients with coagulation abnormalities and without as well. Any deviation from the normal range was considered abnormal in the coagulation parameters recorded in our study.

### Ethical consideration

The study design was approved by the Research Ethics Committee of the Faculty of Medicine at our University. The IRB registration number: Soh-Med-21-07-34. And it was carried following the principles of the 1964 Declaration of Helsinki and its 2013 revision. The study protocol was registered in ClinicalTrials.gov. ID: NCT04982263.

### Statistical methods

Data was analyzed using IBM SPSS Statistics for Windows version 20. Quantitative data was expressed as means ± standard deviation, median and range. Qualitative data was expressed as number and percentage. Quantitative data was tested for normality by Shapiro–Wilk test and normality plots. Independent samples T test was used for normally distributed data. Mann–Whitney U test and Spearman's correlation were used for data which wasn't normally distributed. Chi-square (χ^2^) test and Fisher's Exact Test were used for comparison of qualitative variables as appropriate. Binary logistic regression analysis was used to determine predictor variables of severe cases of COVID-19.

Kaplan–Meier curve and cox regression were used to assess the significance of coagulation abnormalities’ role on patient survival. A 5% level was chosen as a level of significance in all statistical tests used in the study. The figures were performed using the GraphPad Prism software package, version 5.02 (San Diego, CA).

### Patient consent statement

In accordance with the Declaration of Helsinki, all patients provided their informed consent after proper counselling before participation in the study.

## Results

### Baseline characteristics of patients

From January 2021 to June 2021, 267 newly confirmed diagnosed patients with COVID-19 were included in this study. The pooled patients' median age was 37(18–87) years old, with 56.2% of them were men. 57 (21.3%) of the 267 patients had one or more comorbid medical problems. The most prevalent comorbid conditions were diabetes mellitus (DM) (21/267 (7.9%), DM with systemic hypertension (21/267 (7.9%), and hypertension alone (15/267 (5.6%). 71 (26.6%) patients were admitted in ICU. During their ICU stay, 64 of 71 (90%) patients required invasive mechanical ventilation and/or inotropes and vasopressors. Based on the indication to hospitalization, there were 144 patients need hospital admission, and 123 were treated as outpatient management. From the onset of the disease to admission, the median time was 5 days (3–9 days). The study group was divided according to the severity of the disease into 196 (73.4%) non-severe cases and 71(26.5%) severe cases. Significantly, hospitalized and severe patients were older than comparable groups.

The most frequent clinical symptom in the non-severe cases was fever n = 144 (73.5%) while in the severe cases was dyspnea n = 65 (91.5%). Dyspnea was significantly more common in hospitalized and severe groups (P ≤ 0.001).The difference between the groups as regard the presence of DM, hypertension or both diseases was highly significant (P ≤ 0.001). The rural residence was significantly more common in the hospitalized and severe group. Patients' clinical characteristics are showed in Table 1 and supplemental Table S1.

**Table 1:** Demographic, clinical data and outcome among severe and non-severe patients (no. = 267).

Characteristics	Severity		*P* value
	Non-severe (no. = 196)	Severe (no. = 71)	
**Age**			
Mean ± S.D	39.59 ± 15.62	52.65 ± 14.92	< 0.001*
Median (Range)	34 (18–87)	55 (26–85)	
**Sex**			
Male	117 (59.7%)	33 (46.5%)	0.055
Female	79 (40.3%)	38 (53.5%)	
Fever	144 (73.5%)	39 (54.9%)	0.004
Cough	104 (53.1%)	46 (64.8%)	0.088
Dyspnea	70 (35.7%)	65 (91.5%)	< 0.001
Coagulation abnormalities	46 (23.1%)	51 (71.8%)	< 0.001
**Death**			
Died	0 (0.0%)	8 (11.3%)	< 0.001^#^
No	196 (100%)	63 (88.7%)	

P-value was calculated by Chi-Square Test.

*P-value was calculated by Mann Whitney test.

^#^P-value was calculated by Fisher's Exact Test.

P-value < 0.05 is statistically significant.

### Hematological data, inflammatory markers, and coagulation abnormalities

Complete blood count showed statistically highly significant increase in neutrophil count, NLR and reduced hemoglobin (HB) level and lymphocytes in hospitalized and severe cases groups. Thrombocytopenia was detected in 11.6% (31 out of 267) patients. The thrombocytopenia occurrence was higher among older male 74.2% (23 out of 31) patients than female 25.8% (8 out of 31) patients.

Coagulation parameters PC, aPTT, D-Dimer, fibrinogen and AT-III were highly significant deranged in hospitalized and severe group in almost all parameters, same for inflammatory markers; CRP, Serum Ferritin, LDH and ESR (Table 2 and supplemental Table S2).

**Table 2:** The relation between laboratory findings and severity (No. = 267).

Laboratory findings		Severity		P-value
		Non-severe (no. = 196)	Severe (no. = 71)	
WBCs (4–11 × 10^9^/L)	Mean ± S.D	7.59 ± 3.33	7.94 ± 3.74	0.722
	Median (range)	6.9 (2.6–21)	6.9 (2–19.7)	
Neutrophils (45–75%)	Mean ± S.D	65 ± 17.19	69.06 ± 12.56	0.093
	Median (range)	65 (29–96)	71 (30–90)	
Lymphocytes (20–45%)	Mean ± S.D	29.4 ± 16.41	21.23 ± 13.53	< 0.001
	Median (range)	30 (2–66)	18 (3–62)	
NLR (1–3)	Mean ± S.D	4.79 ± 6.6	5.71 ± 5.61	< 0.001
	Median (range)	2.22 (0.45–48)	3.88 (0.48–30)	
HB (F:12–14 g/dl M:14–16 g/dl)	Mean ± S.D	12.97 ± 1.7	11.61 ± 1.74	< 0.001
	Median (range)	13.1 (8–16.5)	11.7 (8.2–15.6)	
MCV (77–96 fl)	Mean ± S.D	80.33 ± 6.66	77.14 ± 5.81	< 0.001
	Median (range)	81.7 (60–95.4)	77.3 (64.5–87.8)	
MCH (26–32 pg)	Mean ± S.D	24.88 ± 3.1	24.17 ± 2.55	0.093
	Median (range)	25 (18–31.6)	24 (18–30.1)	
PLT (150–400 × 10^9^/L)	Mean ± S.D	267.91 ± 79.34	245.06 ± 118.36	0.494
	Median (range)	258 (30–534)	271 (31–448)	
Ferritin (30–160 ng/mL)	Mean ± S.D	275.08 ± 162.31	445 ± 260.65	< 0.001
	Median (range)	253.67 (11.9–1105.23)	363 (158–1295)	
LDH (100–190 IU/L)	Mean ± S.D	346.63 ± 123.82	608.61 ± 242.81	< 0.001
	Median (range)	339.5 (75–685)	578 (156.3–1553)	
D-dimer (0–0.5 mg/L)	Mean ± S.D	0.65 ± 0.78	3.43 ± 2.5	< 0.001
	Median (range)	0.3 (0.1–4.8)	2.7 (0.34–14.01)	
CRP (< 6 mg/L)	Mean ± S.D	14.75 ± 17.98	39.62 ± 53.7	< 0.001
	Median (range)	6 (0–96)	24 (3–384)	
PC (70–120%)	Mean ± S.D	85.75 ± 10.97	76.27 ± 17.5	< 0.001
	Median (range)	85 (34.5–110)	80 (30.3–100)	
aPTT (25–31 s)	Mean ± S.D	30.98 ± 5.2	42.08 ± 17.01	< 0.001
	Median (range)	30.5 (24.5–82)	34.1 (24.5–97.5)	
AT-III (80–120%)	Mean ± S.D	90.33 ± 9.57	79.11 ± 10.2	< 0.001
	Median (range)	90 (60–118)	78 (50–97)	
Fibrinogen (2–4 g/L)	Mean ± S.D	3.61 ± 0.86	4.49 ± 0.9	< 0.001
	Median (range)	3.59 (2–5.7)	4.66 (0–6.1)	
ESR	Mean ± S.D	22.02 ± 14.71	43.24 ± 24.12	< 0.001
	Median (range)	18 (6–130)	42 (5–150)	

*WBCs* white blood cells, *Hb* hemoglobin, *NLR* neutrophil-to-lymphocyte ratio, *MCV* mean corpuscular volume, *MCH* mean corpuscular hemoglobin, *PLT* platelets, *LDH* lactate dehydrogenase, *ESR* erythrocyte sedimentation rate, *CRP* C-reactive protein, *PC* prothrombin concentration, *aPTT* activated partial thromboplastin, *AT-III* antithrombin-III.

P-value was calculated by Mann Whitney test.

P-value < 0.05 is statistically significant.

**Figure 1:**
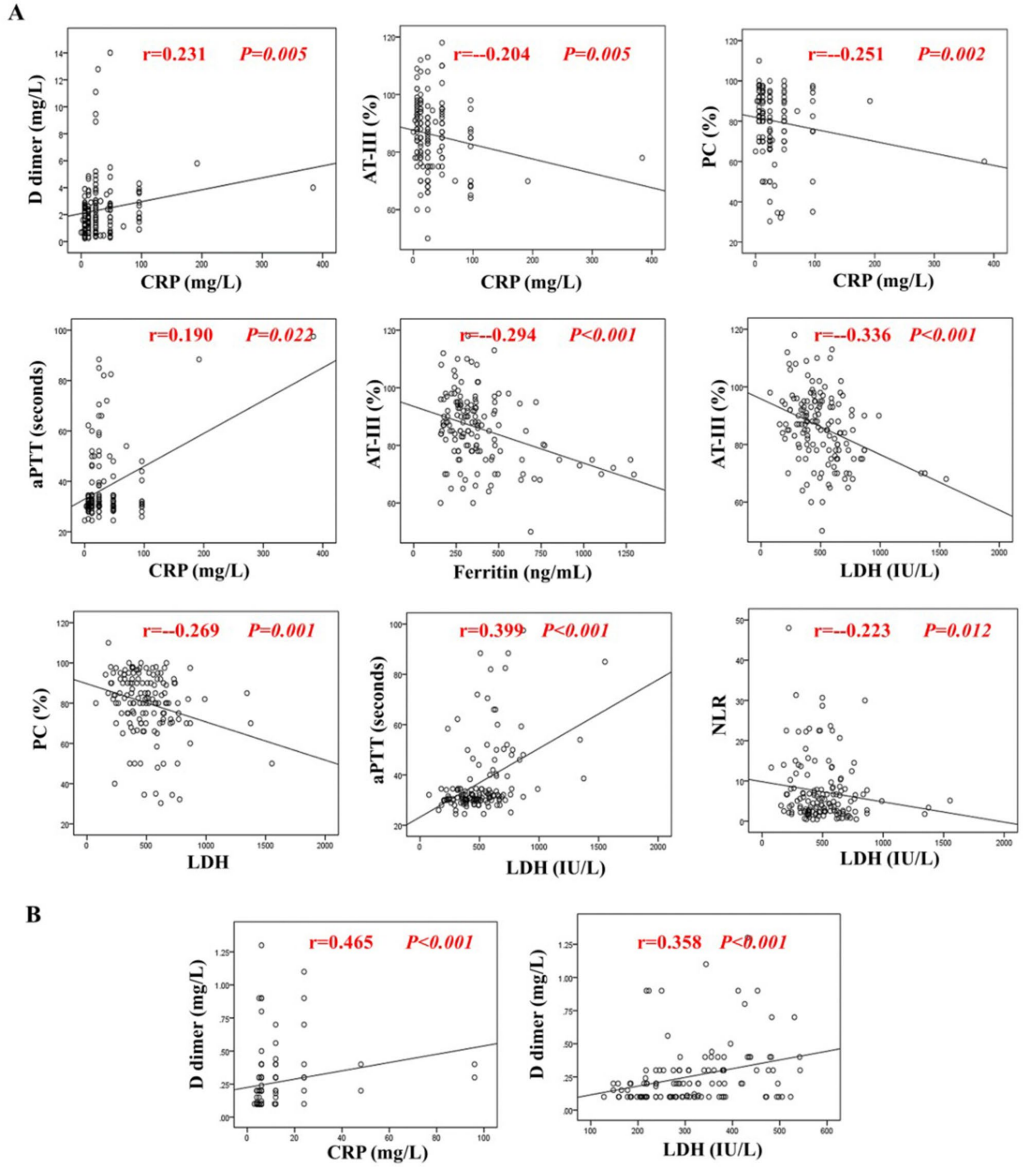
Schematic box plots representing the correlations of coagulation parameters (PC, aPTT, Fibrinogen, AT-III, and d-dimer) with inflammatory parameters (CRP, ferritin, NLR and LDH) among hospitalized (144) cases (**A**), and non- hospitalized (123) cases (**B**). *LDH* lactate dehydrogenase, *CRP* C-reactive protein, *PC* prothrombin concentration, *aPTT* activated partial thromboplastin, *ATT-III* antithrombin-III, *NLR* neutrophil-to-lymphocyte ratio, *r* Spearman rank correlation coefficient, *P* P value.

In hospitalized group (144 patients) there were significant negative correlations between PC and serum ferritin, CRP, and LDH, and significant positive correlations between aPTT and CRP, and LDH were detected. We observed significant negative correlations between AT-III and serum ferritin, CRP, LDH and NLR. While significant positive correlations between D dimer and CRP, and LDH were detected (Supplemental Table S3 and Fig. 1A) while in the non- hospitalized group (123 patients) there was highly significant positive correlation between the level of D-dimer with CRP and LDH level (Supplemental Table S4 and Fig. 1B).

We observed among severe group significant negative correlations between PC/AT-III and serum ferritin, CRP, ESR and LDH, but significant positive correlations between aPTT/fibrinogen/D dimer and serum ferritin, CRP, ESR and LDH were detected among severe group (Table 3 and Fig. S1).

**Table 3:** Correlation between coagulation parameters and inflammatory markers in severe group.

Hematological and inflammatory statistics	PC		a PTT		Fibrinogen		AT-III		D dimer	
	r	P-value	r	P-value	r	P-value	r	P-value	r	P-value
LDH	− 0.287	< 0.001	0.357	< 0.001	0.265	< 0.001	− 0.191	0.002	0.565	< 0.001
ESR	− 0.242	< 0.001	0.292	< 0.001	0.372	< 0.001	− 0.220	< 0.001	0.553	< 0.001
CRP	− 0.265	< 0.001	0.251	< 0.001	0.334	< 0.001	− 0.144	0.018	0.616	< 0.001

*LDH* lactate dehydrogenase, *ESR* erythrocyte sedimentation rate, *CRP* C-reactive protein, *PC* prothrombin concentration, *aPTT* activated partial thromboplastin, *AT-III* antithrombin-III.

r = spearman correlation coefficient.

P-value < 0.05 is statistically significant.

The univariate and multivariate logistic regression analysis revealed that the prediction of the D-dimer, AT-III and LDH can be utilized for excellent independently predicting the severity risk of COVID-19 (Tables 4, 5).

**Table 4:** Multiple binary logistic regression analysis of predictor variables of severe illness.

Variables	OR (CI_95%_)	P-value
PC	1.02 (0.98–1.07)	0.373
aPTT	1.04 (0.97–1.11)	0.288
Fibrinogen	1.7 (0.93–3.09)	0.084
D-dimer	5.54 (3.13–9.81)	< 0.001
AT-III	0.91 (0.85–0.96)	0.001
Ferritin	1 (1–1)	0.796
LDH	1.01 (1–1.01)	< 0.001

*LDH* lactate dehydrogenase, *PC* prothrombin concentration, *aPTT* activated partial thromboplastin, *ATT-III* antithrombin-III.

*Statistically significant.

**Table 5:** Final binary logistic regression analysis of predictor variables of severe illness.

Variables	OR (CI_95%_)	P-value
D-dimer	5.95 (3.44–10.3)	< 0.001
AT-III	0.89 (0.84–0.95)	< 0.001
LDH	1.008 (1.004–1.01)	< 0.001

*LDH* lactate dehydrogenase, *ATT-III* antithrombin-III.

*Statistically significant.

**Figure 2:**
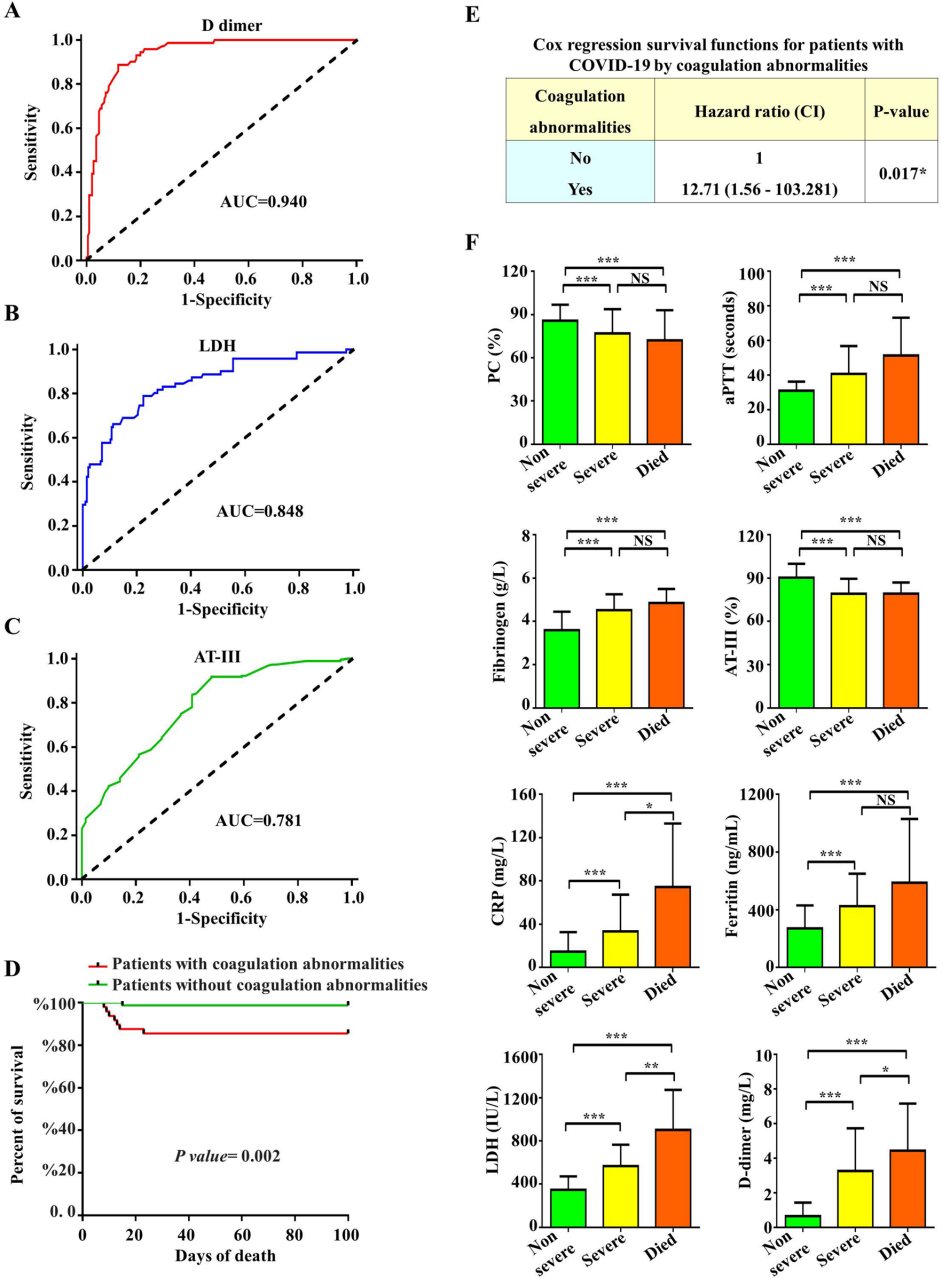
Receiver operator characteristic curves for D-dimer (**A**), LDH (**B**), and AT-III (**C**) levels of COVID-19 patients in non-severe VS severe cases. (**D**) Kaplan–Meier estimator for comparison of survival functions for patients with COVID-19 by coagulation abnormalities. (**E**) Cox regression survival functions for patients with COVID-19 by coagulation abnormalities. (**F**) Characteristics of coagulation parameters and inflammatory biomarkers in death and survival group (non- severe and severe cases). Asterisks indicate significant P value. * indicated P value < 0.05, ** indicated P value < 0.01, *** indicated P value < 0.001. *LDH* lactate dehydrogenase, *PC* prothrombin concentration, *aPTT* activated partial thromboplastin, *ATT-III* antithrombin-III, *CI* confidence interval.

The ROC curves and area under the curve (AUC) demonstrated that D dimer (AUC = 0.940), LDH (AUC = 0.848), and AT-III (AUC = 0.781), had good predictive ability in severe VS non-severe cases [95 percent confidence interval (95 percent CI) = 0.72–0.84], [95% CI 0.79–0.90] and 0.94 [95% CI 0.91–0.97] respectively (all p < 0.001, Fig. 2A–C).

With a cut-off of more than 2.0 ng/L, the sensitivity and specificity of D dimer in predicting severity were 76% and 93%, respectively. With a cut-off of greater than 500 IU/L, the sensitivity and specificity of LDH in predicting severity were 65% and 90%, respectively. With a cut-off of less than 75%, the sensitivity and specificity of ATIII in predicting severity were 90% and 60%, respectively.

### Coagulation and inflammatory parameters of COVID-19 in death and survival cases

Figure 2D,E show the significance of coagulation abnormalities’ role on patient survival using Kaplan–Meier curve and cox regression assessment.

Despite aggressive therapy, patients with coagulation abnormalities had a higher death rate (7.2%) (7 out of 97 cases), whereas patients without coagulation abnormalities had only one death (0.59%) (1 out of 170 cases). Levels of coagulation parameters and inflammatory biomarkers groups (aPTT, fibrinogen, CRP, LDH, serum ferritin, and DD levels) were considerably greater in the COVID-19 death group than in the non-severe groups, although PC and AT-III levels were significantly lower. The levels of PC, aPTT, AT-III, fibrinogen and serum ferritin did not differ significantly among the non-survival group and the severe group (Fig. 2F). The D dimer proved to be excellent in independently predicting mortality risk.

## Discussion

The extreme inflammatory condition caused by COVID-19 causes hemostasis to be severely disrupted and coagulation parameters to be significantly altered^8,9^. COVID-19 infection has been linked to impairment of coagulation parameters as well as a high rate of venous and arterial thrombotic complications, including unusual thrombotic events^10^.

In COVID-19 patients, coagulopathy is common^11–13^ and has been linked to poorer outcomes^12,14,15^. Tang and colleagues^2^, for example, found that in COVID-19 patients, abnormal conventional coagulation tests, particularly notably high D-dimer and fibrinogen levels after hospitalization, were linked to a poor prognosis.

The goal of this study was to see how well coagulation markers predicted COVID-19 patients' hospitalization, disease severity and fatality.

We found in this prospective single-center study, that hospitalized patients with SARS-CoV-2 infection had a marked hypercoagulability state, characterized by elevated aPTT, NLR, D dimer and plasma fibrinogen levels, with decreased PC, and AT-III plasma levels. According to the disease severity, the severity of coagulation abnormalities appears to be related to the severity of inflammation and degrees of COVID-19 severity. Many studies showed that High NLR was significantly associated with increasing the risk of thrombosis, the disease severity and mortality in COVID-19^16,17^.

The existence of a lupus anticoagulant (LA) or antiphospholipid antibodies (APA) is a possible explanation for the prolongation of the aPTT in some COVID-19 patients. In a series of 56 hospitalized patients with COVID-19, LA was detected in 44.6%^18^. We previously also observed a 20.7% prevalence of transient APA in 29 COVID-19 patients with unusual thrombotic events^10^.

In the clinic, PC and prothrombin time (PT) are commonly used to measure coagulation function as one of the most critical markers. Many studies have looked at how PC and PT changes in COVID-19 patients^19–21^. Several studies^21–23^ found that severe patients had significantly decrease in PC and longer PT than non-severe patients. Furthermore, non-survivors showed greater PT levels and lower PC than survivors in COVID-19 trial^2^. We also discovered that severe patients with COVID-19 and non-survivors had considerably lower admission PC than non-severe cases.

Furthermore, the D-dimer level also has been the topic of many COVID-19 investigations. According to a meta-analysis, 37.2 percent of COVID-19 patients had a high D-dimer level^24^. Many researchers suggested that D-dimer could be a useful early indicator for improving COVID-19 patient management^24,25^. They found that COVID-19 patients with D-dimer levels greater than 2.0 g/mL had a higher mortality rate than those who have D-dimer levels less than 2.0 g/mL. Also, high D-dimer levels were used as an excellent marker for identifying groups at high risk of thromboembolism in severe COVID-19 patients presented with pneumonia patients^20^.

Our study showed that D-dimer levels were greater than the upper limit of normal in severe and hospitalized patients with COVID-19, and non-survivors exhibited significantly higher D-dimer levels than survivors. The same findings were noticed regarding to fibrinogen levels. Zou et al.^26^ studied the coagulation function of 303 COVID-19 patients and discovered a correlation between increased fibrinogen and the disease severity.

Regarding of AT-III, we discovered that the median value is lower in severe cases than in non-severe cases, with medians of 78 and 90, respectively. Anakl et al. and Arachchillage et al. discovered similar results^27,28^. Interestingly, in recent studies, an acquired transient deficiency of AT-III was reported^10,29^.

Moreover, we observed that marked coagulation abnormalities such as higher aPTT, D- dimer, and fibrinogen levels were more frequently detected in non-survivals compared to non-severe.

Blood coagulation abnormalities were shown to be common in COVID-19 patients who were hospitalized or in intensive care units, and these coagulation abnormalities were statistically significant correlated with multi-inflammation factors among the subcategories based on hospitalization as well as disease severity status akin to Lui et al.^30^ and Pujani et al.^31^.

A retrospective cohort study conducted in Spain showed that COVID-19 non-survivors had significantly abnormal coagulation than survivors^32^, indicating that coagulation parameters could be an efficient measure for predicting the prognosis of patients with SARS COV-2 and used as guiding clinical management. Long et al. also found that coagulation dysfunction is a likely finding in severe and critically ill patients. D-dimer, prothrombin time and concentration were also found to be important indicators in prediction of COVID-19 patient mortality in many studies^11,12^.

Furthermore, patients with severe COVID-19 had greater amounts of inflammatory biomarkers and many reports suggested they can be used as prognostic markers^33,34^. Similarly, we found that most hospitalized patients with severe COVID-19 infection exhibited a pronounced inflammatory state, characterized by a higher level of inflammatory markers including CRP, ESR, ferritin, LDH, and NLR. In addition, we observed lower hemoglobin levels and platelets count with lymphopenia, and neutrophilia among severe cases. Lymphopenia could be explained by Covid- 19, which enters human cells by attaching to the receptor angiotensin-converting enzyme 2 (ACE2), which is found on many cells and tissues. As a result of the presence of the receptor angiotensin-converting enzyme 2 (ACE2) on lymphocytes surfaces, it became more vulnerable to virus invasion and lysis. This conclusion could be confirmed by a considerable drop in blood lymphocytes within a week of COVID-19 infection^35^.

In the current investigation, multivariate logistic regression analysis revealed that the prediction of the D-dimer, LDH and AT-III can be contemplated as excellent independently predictors of the severity risk in COVID-19, with a cut off (2.0 ng/L, 500 IU/L, and 75%) and AUC of (0.940, 0.848, 0.781) respectively by ROC curve analysis. Chen et al.^36^ discovered that D-dimer levels more than 1 g/mL on admission were associated with an increased risk of in-hospital death. Cui et al.^20^ reported that increasing D-dimer levels at admission were strongly linked with death using multivariable logistic regression. Wu et al.^14^ discovered that D-dimer associated with the progression from ARDS to mortality in a bivariate Cox regression analysis. These results were in a line with Liu et al.^13^ report and another COVID-19 study that found the AUCs of PT and D-dimer for predicting fatality at admission were 0.643 and 0.742, respectively^12^.

Given these data, we believe that specific coagulation function parameters upon admission, such as D-dimer, AT-III, and fibrinogen, in addition to serum ferritin and LDH may reliably predict the fate of COVID-19 patients. Although serial measurements may offer greater information and guide treatment. Assessing coagulation parameters at admission has the benefit of enabling doctors with essential information at the right time and helping doctors in providing valuable treatments quickly in the early stages of hospital treatment.

In conclusion: because most COVID-19 patients have coagulopathy, coagulation systems have value. Early assessment and dynamic monitoring of coagulation system parameters may be a benchmark in the control of COVID-19 severity and death by adding another indication to the criteria for hospitalization and preventing or stopping the occurrence of thrombus or DIC in COVID-19 patients.

## Supplementary Information

Supplementary Information.

## Data Availability

The authors confirm that all data supporting the findings of this study are available within the article, its supplementary material, and upon reasonable request. All supporting data are available within the article.
