# Supplementary material for: Coagulation parameters abnormalities and their relation to clinical outcomes in hospitalized and severe COVID-19 patients: prospective study
Source: Sci Rep. 2022 Aug 1;12:13155. doi: 10.1038/s41598-022-16915-8 (PMC9340692; doi:10.1038/s41598-022-16915-8)
Supplement: Supplementary file 1 — Supplementary Information. [file 41598_2022_16915_MOESM1_ESM.docx]

**Supplemental data**

**Figure S1.** Schematic box plots representing the correlations of coagulation parameters (PC, aPTT, Fibrinogen, AT-III, and D-dimer) with inflammatory parameters (LDH, ESR and CRP) among Non-severe (196) cases (blue colour), and Severe (71) cases (red colour).

**Abbreviations:** LDH; lactate dehydrogenase, ESR; erythrocyte sedimentation rate, CRP; C-reactive protein, PC; prothrombin concentration, aPTT; activated partial thromboplastin, ATT-III; antithrombin-III.


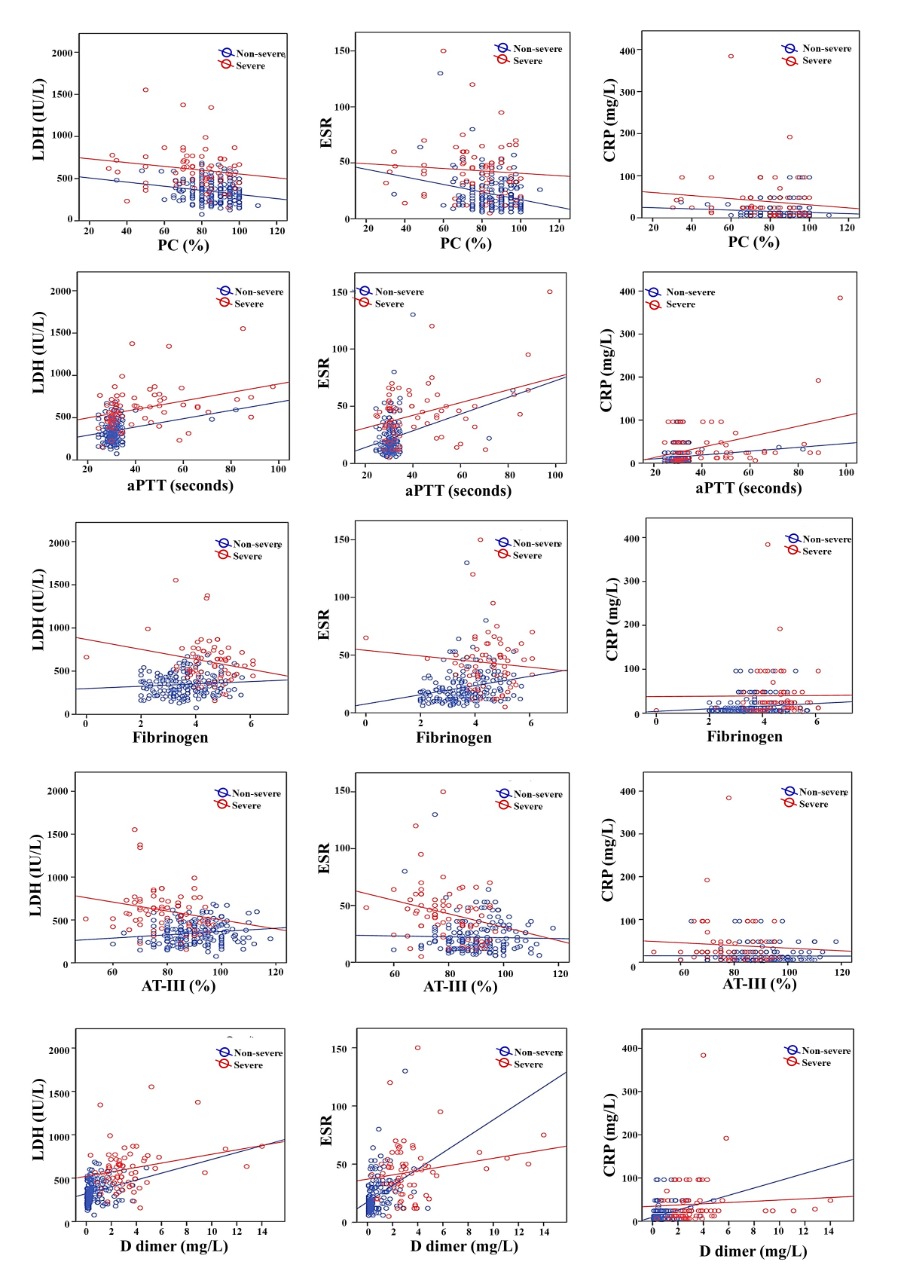


**Table (S1): Demographic, clinical data and outcome among hospitalized and non-hospitalized patients (No.=267)**

| **Characteristics** | **Hospitalization** | | **P-value** |
| --- | --- | --- | --- |
|  | **Hospitalized**  **(No.=144)** | **Non hospitalized**  **(No.=123)** |  |
| **Age**  Mean± S.D.  Median (Range) | 52.94 ± 15.68  54 (22 – 87) | 31.5 ± 7.24  31 (18 – 64) | **<0.001*** |
| **Sex**  Male  Female | 77 (53.5%)  67 (46.5%) | 73 (59.3%)  50 (40.7%) | 0.335 |
| **Coagulation abnormalities** | 87 (60.4%) | 10 (8%) | **<0.001** |
| **Fever** | 85 (59%) | 98 (79.7%) | **<0.001** |
| **Cough** | 87 (60.4%) | 63 (51.2%) | 0.131 |
| **Dyspnea** | 107 (74.3%) | 28 (22.8%) | **< 0.001** |
| **Comorbidities**  DM  DM/HTN  HTN  None | 21 (14.6%)  21 (14.6%)  15 (10.4%)  87 (60.4%) | 0 (0.0%)  0 (0.0%)  0 (0.0%)  123 (66.7%) | **< 0.001** |
|  |  |  |  |
| **Severity**  Non-severe  Severe | 73 (50.7%)  71 (49.3%) | 123 (100%)  0 (0.0%) | **< 0.001** |
| **Death**  Died  No | 8 (5.6%)  136 (94.4%) | 0 (0.0%)  123 (100%) | **0.007#** |
|  |  |  |  |

**P-value was calculated by Chi-Square Test**

*** P-value was calculated by Mann Whitney test**

**#P-value was calculated by Fisher's Exact Test**

**P- value <0.05 is statistically significant.**

**Table (S2): The relation between laboratory findings and hospitalization status (N=267)**

| **Laboratory findings** | | **Hospitalization** | | **P-value** |
| --- | --- | --- | --- | --- |
|  |  | **Hospitalized**  **(No.=144)** | **Non hospitalized**  **(No.=123)** |  |
| **WBCs**  (4-11x 10^9^/L) | Mean± S.D.  Median (Range) | **7.88 ± 3.51**  **6.9 (2 – 21)** | **7.46 ± 3.36**  **7.03 (2.6 – 18.9)** | **0.412** |
| **Neutrophils**  (45-75%) | Mean± S.D.  Median (Range) | **72.9 ± 14.56**  **75 (30 – 96)** | **58.09 ± 14.22**  **58 (29 – 93)** | **< 0.001** |
| **Lymphocytes**  (20-45%) | Mean± S.D.  Median (Range) | **20.91 ± 15.49**  **16 (2 – 66)** | **34.62 ± 13.44**  **36 (5 – 63)** | **< 0.001** |
| **NLR**  (1-3) | Mean± S.D.  Median (Range) | **7.27 ± 7.7**  **4.53 (0.45 – 48)** | **2.41 ± 2.38**  **1.61 (0.46 – 18.6)** | **< 0.001** |
| **HB** ( F:12-14 g/dl M:14-16 g/dl ) | Mean± S.D.  Median (Range) | **12.28 ± 1.99**  **12.4 (8 – 16.5)** | **12.99 ± 1.5**  **13.1 (8.6 – 16.1)** | **0.002** |
| **MCV**  (77-96 fl) | Mean± S.D.  Median (Range) | **78.42± 7.25**  **78.7 (60.7 – 93)** | **80.73 ± 5.49**  **82 (60 – 95.4)** | **0.006** |
| **MCH**  (26-32 pg) | Mean± S.D.  Median (Range) | **24.89 ± 3.07**  **24.8 (18 – 31.6)** | **24.45 ± 2.85**  **24.2 (18 – 31)** | 0.236* |
| **PLT**  (150-400×10^9^/ L) | Mean± S.D.  Median (Range) | **261.21 ± 109.84**  **270 (30 – 508)** | **262.57 ± 64.66**  **258 (83 – 534)** | **0.478** |
| **Ferritin**  (30–160 ng/mL) | Mean± S.D.  Median (Range) | **379.63 ± 215.1**  **320 (158 – 1295)** | **250.76 ± 173.67**  **227.25 (11.9 – 1105.23)** | **< 0.001** |
| **LDH**  (100 to 190 IU/L) | Mean± S.D.  Median (Range) | **504.21 ± 220.96**  **475 (75 – 1553)** | **313.38 ± 103.07**  **296 (128 – 543)** | **< 0.001** |
| **D-dimer**  (0–0.5mg/L) | Mean± S.D.  Median (Range) | **2.36 ± 2.15**  **1.83 (0.24 – 14.01)** | **0.26 ± 0.23**  **0.2 (0.1 – 1.3)** | **< 0.001** |
| **CRP**  ( < 6 mg/L) | Mean± S.D.  Median (Range) | **31.3 ± 41.39**  **24 (0 – 384)** | **9.74 ± 13.22**  **6 (3 – 96)** | **< 0.001** |
| **PC**  ( 70%–120%) | Mean± S.D.  Median (Range) | **80.07 ± 15.7**  **82.25 (30.3 – 110)** | **86.93 ± 9.6**  **85 (60 – 100)** | **< 0.001** |
| **aPTT**  (25–31 seconds) | Mean± S.D.  Median (Range) | **37.04 ± 14.1**  **31.5 (24.5 – 97.5)** | **30.29 ± 2.04**  **30.3 (24.5 – 34.6)** | **< 0.001** |
| **AT-III**  ( 80%–120%) | Mean± S.D.  Median (Range) | **86.05 ± 12.07**  **87 (50 – 118)** | **88.86 ± 9.22**  **90 (60 – 110)** | **0.033*** |
| **Fibrinogen**  (2–4 g/L) | Mean± S.D.  Median (Range) | **4.27 ± 0.84**  **4.29 (0 – 6.1)** | **3.34 ± 0.83**  **3.3 (2 – 5.7)** | **< 0.001** |
| **ESR** | Mean± S.D.  Median (Range) | 37.7 ± 22.2  34.5 (5 – 150) | 15.9 ± 6.03  15 (6 – 33) | < 0.001 |

Abbreviation: WBCs; white blood cells. Hb; hemoglobin; neutrophil-to-lymphocyte ratio; MCV, mean corpuscular volume. MCH; mean corpuscular hemoglobin, PLT; platelets, LDH; lactate dehydrogenase; ESR; erythrocyte sedimentation rate, CRP: C-reactive protein, PC; prothrombin concentration, aPTT; activated partial thromboplastin, ATT-III; antithrombin-III.

**P-value was calculated by Mann Whitney test**

***P-value was calculated by Independent Samples T test**

**P- value <0.05 is statistically significant.**

**Table (S3): Correlation between coagulation parameters and inflammatory markers in hospitalized COVID -19 patients** **(n=144)**

|  | **CRP** | | **Ferritin** | | **LDH** | | **NLR** | |
| --- | --- | --- | --- | --- | --- | --- | --- | --- |
|  | **r** | **P-value** | **r** | **P-value** | **r** | **P-value** | **r** | **P-value** |
| **D dimer** | 0.231 | **0.005** | 0.135 | 0.108 | 0.328 | **< 0.001** | -0.141 | 0.091 |
| **AT-III** | -0.204 | **0.014** | -0.294 | **< 0.001** | -0.336 | **< 0.001** | -0.227 | **0.006** |
| **Fibrinogen** | 0.067 | 0.424 | 0.142 | 0.09 | 0.006 | 0.943 | 0.003 | 0.968 |
| **PC** | -0.251 | **0.002** | -0.177 | **0.034** | -0.269 | **0.001** | -0.037 | 0.658 |
| **aPTT** | 0.190 | **0.022** | 0.119 | 0.154 | 0.399 | **< 0.001** | -0.095 | 0.256 |

Abbreviation: LDH; lactate dehydrogenase; PC; prothrombin concentration, aPTT; activated partial thromboplastin, ATT-III; antithrombin-III.

**r = spearman correlation coefficient**

**P-value <0.05 is statistically significant**

**Table (S4): Correlation between coagulation parameters and inflammatory markers in in non-hospitalized COVID -19 patients (n=123)**

|  | **CRP** | | **Ferritin** | | **LDH** | | **NLR** | |
| --- | --- | --- | --- | --- | --- | --- | --- | --- |
|  | **r** | **P-value** | **r** | **P-value** | **r** | **P-value** | **r** | **P-value** |
| **D dimer** | 0.465 | **< 0.001** | 0.125 | 0.167 | 0.358 | **< 0.001** | 0.068 | 0.454 |
| **AT-III** | 0.091 | 0.318 | 0.062 | 0.497 | 0.135 | 0.136 | -0.039 | 0.665 |
| **Fibrinogen** | 0.023 | 0.803 | -0.120 | 0.185 | -0.006 | 0.947 | -0.108 | 0.235 |
| **PC** | -0.090 | 0.323 | -0.054 | 0.556 | -0.141 | 0.121 | -0.010 | 0.917 |
| **a PTT** | 0.001 | 0.987 | 0.134 | 0.139 | 0.126 | 0.166 | -0.039 | 0.668 |

Abbreviation: LDH; lactate dehydrogenase; PC; prothrombin concentration, aPTT; activated partial thromboplastin, ATT-III; antithrombin-III.

**r = spearman correlation coefficient**

**P-value <0.05 is statistically significant**
